# Supplementary material for: Patterns of Intron Gain and Loss in Fungi
Source: PLoS Biol. 2004 Nov 30;2(12):e422. doi: 10.1371/journal.pbio.0020422 (PMC532390; doi:10.1371/journal.pbio.0020422)
Supplement: Table S1 — Also available at http://genes.mit.edu/NielsenEtAl/. (4.3 MB ZIP). [file pbio.0020422.st001.zip › NielsenEtAl/html/579.html]

AN3057.1.NCU09710.1.MG03858.1.FG06053.1


```
 CLUSTAL W (1.82) Multiple Sequence Alignments - Introns Inserted


Sequence 1: NCU09710.1	557 aa
Sequence 2: MG03858.1	551 aa
Sequence 3: FG06053.1	531 aa
Sequence 4: AN3057.1	554 aa
Alignment Length: 613 aa
Number Identitical Residues: 134 aa
Alignment Score (without introns) 7615


MG03858.1 	MRLSAELAVLASSLWILVGAEPAPTDQQCSPFPGVKG-TVTTCDKPQ-QIAIIG1AGAAG
NCU09710.1	MRLPCDLQSAATALLLFI----SPALARRIPDDVIEGDNVDDRETPVRQVAIIG1AGAAG
FG06053.1 	MYFSTTFGLAFIASLLSVP---------------CEAETVNN-------VAIIG1AGAAG
AN3057.1  	-MEPFLRGNLRWCLWIWALCYATAVAFNPLSKLRGTQSHLNSVEAGPKQVAVIG1AGAAG
          	   .        .  :   . .:.      .      . :   .    .:*:** *****

MG03858.1 	ASAAYHLTQFASDANIPINITVFEKTTRIGGRTLTVNAYDDPEHPVELGASIFIELNHIL
NCU09710.1	ASAAYHLRKYADQYNVNVNITIFEKTDHIGGRSLTINPYDDPSLRMELGASIFIEKNYIL
FG06053.1 	SSAAFHLRKYALEENIAINITVFEKTDHVGGRSLTVPAYNDPSLPIELGASIFVGANHIL
AN3057.1  	ASTAYSLRKYADSFGIPVNITVFERASYIGGRSTTVNVFDDPAYPIELGASIFVQVNYNL
          	:*:*: * ::* . .: :***:**::  :***: *:  ::**   :*******:  *: *

MG03858.1 	YNAANHFKLPLK--TPG-PEEDEYLVIWDGSRFRFESRDSQWNWWKLVKLFWK~YG-QAP
NCU09710.1	NQSVEEFGLRKK--DPD-VGSDPKMGIWDGENFVFEIDTSSSWISQLWHIVWK~YGIRTP
FG06053.1 	VNTSRQFQLPLS--KPHRLDKDDVTAIWDGIDFVFQTTEGSWGGWDLAKMFWS2MIGEFL
AN3057.1  	VNATKELGLDVRGASTERPGDTAAIGVWDGKEFVFVMEDSSS-WWNIAKLLWR~YG-LSP
          	 :: ..: *   .: .    .     :***  * *    ..    .: ::.*        

MG03858.1 	YKAQQLMKATIAEFLKLYEEPYFPFRSLTTTAYELGL~TKITGLTGTQYLDQN~N0IGSS
NCU09710.1	KRTNDLVQETVSKFLR--------------------I2AQIYG----------~-~---A
FG06053.1 	KLYEDPYFPFKSLSQR---------------AEQLGL~NRFTSLTGEQVLKNA0N~IDPR
AN3057.1  	VRAQNLMKGTVNKFLQLYQEPLFPFKSLTAAAASVGL~LDATSTTGATFLEKN~N~VSPG
          	   ::          :  ...  .  : :::: . .:     . :.    ..  .  .. 

MG03858.1 	FSEDVIQAATRVNYASNLGRIHGLDAM0VSIAPDGASQVTGGNWQIFDHMLKATNASLHL
NCU09710.1	YAHDIVQASTRVNYATNIGRLHGLDTM~VAMAPAGATAVQGGNWQIFQKMVERSGATIML
FG06053.1 	FAREILQVATRVNYASNLAYIHGLETL0VSLATDNAMSVETGNWRIFDEMIMDSGAAVYR
AN3057.1  	FSRDLIQASTRVNYGQNLPLIHGLETM~VCLATDGAVAVEGGNWRIFEGMLKSARANVQL
          	::.:::*.:*****. *:  :***::: *.:*. .*  *  ***:**: *:  : * :  

MG03858.1 	NTTVTSIEFDKAD-DDSSILGYVLETSKDSSGSIASS--SQRFDKVVIATPYQFSNIHTG
NCU09710.1	NTSVSSISFAKTGSEDTTSAKYSLRAIHSSSQEDTSY--PVEFDNVILANPHQFSNIDIA
FG06053.1 	NLTVASIEKSKTKAQSSSSSKYIITTADAHSKDDTAENYGVEFDNVIIANPWQFSNIKAG
AN3057.1  	NHTVTSITQNKDK-----SVTLDFKDEQSIKQQ------RADFDEVVIAAPYQYSGINIS
          	* :*:**   *            :   .  . .         **:*::* * *:*.*. .

MG03858.1 	EEVLQRPIDEIPYVKLHVTLFTSPLRLSPAFFNLPADAEAP-TTVLTTLGEGDDASS---
NCU09710.1	DGVLETHIDEIPYVRLHVTIFTSPFRYSPGFFGLENPKALP-GTVLTTLGKTDNATSGVA
FG06053.1 	KGVLDRVIDEIPYTKLHVTLFTSSLELNPEFFSMKPGSKAP-ATVYTTLAEGDEAGQGAD
AN3057.1  	P-APKHTPDTIPFHTLHVTLFSSPHRLSGKYFNLKNPSDIPPETILTTLPLGSDLGNAEE
          	  . .   * **:  ****:*:*. . .  :*.:      *. *: ***   .:  ..  

MG03858.1 	GAGKAGFFSISNQRSLTNPATGK---------TEFIYKIFSPEKVTTEFMSSLLGVQVPE
NCU09710.1	GAGSAGFFSISMLRKLVNPKTQK---------EEYAYKIFSPEKVTPEFLSRLFGVKVPE
FG06053.1 	GVGRTGFYSISTLKYVTNPKTGQ---------RERVYKIFSPKVVTAEFLTRILGTEIPE
AN3057.1  	GVGPAAFWSISTLRTVKAPSTSNPPEDRESETKHYVYKIFSPTRPNEAFIREILGLEQKK
          	*.* :.*:***  : :  * * :.... .:.: .  ******   .  *:  ::* :  :

MG03858.1 	SVTAQRTESEAKSWKGTGPVTWYYPHVFYSYPQALPRVTFQDPILGPGLYYTSGMESFIS
NCU09710.1	TFIAEPND---QSSAEVSPISWYYPHVFYSYPKAEPRVTFQDPIVGAGFYYTSGMESFIS
FG06053.1 	PVVS--------GKKQSEHISWYYPHSFYAYPIELPRVTFEDPVIGKGVYYTSGIESFIS
AN3057.1  	IKKAG-----YITDLPKEDISWSYEKLWHPYPFLYPRVTFEDPLLAPGIWYTGGIESFIS
          	   :               ::* * : ::.**   *****:**::. *.:**.*:*****

MG03858.1 	TMETNALMGKNVARLVVDSLLG-----------------------HKADVGEDKDRVVIG
NCU09710.1	TMETNALMGKNVARLIVDDMRGVESGGVQSHDGSLAPGQQLKMTCDRAGRGAGKGGVRKA
FG06053.1 	CMETSAFMGRNVARLVVDNFAGISR-----------PDEPLGAGNEKGPLGEEMEGIKVQ
AN3057.1  	TMETSALMGKNVAALMSQGWLDKG-------------------FVDSPVVEDELGRTEL-
          	 ***.*:**:*** *: :.  .                       .              

MG03858.1 	---------EL--------
NCU09710.1	GEVPKTRPTELPEMPLADL
FG06053.1 	AEG------EL--------
AN3057.1  	-------------------
          	
```
